# Developmental Programming of Cardiovascular Disease Following Intrauterine Growth Restriction: Findings Utilising A Rat Model of Maternal Protein Restriction

**DOI:** 10.3390/nu7010119

**Published:** 2014-12-29

**Authors:** Vladislava Zohdi, Kyungjoon Lim, James T. Pearson, M. Jane Black

**Affiliations:** 1 Department of Anatomy and Developmental Biology, Monash University, Melbourne, VIC 3800, Australia; E-Mails: vladislava.zohdi@monash.edu (V.Z.); jane.black@monash.edu (M.J.B.); 2 Neuropharmacology Laboratory, Baker IDI Heart and Diabetes Institute, P.O. Box 6492 St Kilda Rd Central, Melbourne 8008, Australia; E-Mail: joon.lim@bakeridi.edu.au; 3 Department of Physiology, Monash University, Melbourne, VIC 3800, Australia; 4 Monash Biomedical Imaging, Monash University, Melbourne, VIC 3800, Australia

**Keywords:** diabetes, heart, IUGR, hypertension, maternal diet

## Abstract

Over recent years, studies have demonstrated links between risk of cardiovascular disease in adulthood and adverse events that occurred very early in life during fetal development. The concept that there are embryonic and fetal adaptive responses to a sub-optimal intrauterine environment often brought about by poor maternal diet that result in permanent adverse consequences to life-long health is consistent with the definition of “programming”. The purpose of this review is to provide an overview of the current knowledge of the effects of intrauterine growth restriction (IUGR) on long-term cardiac structure and function, with particular emphasis on the effects of maternal protein restriction. Much of our recent knowledge has been derived from animal models. We review the current literature of one of the most commonly used models of IUGR (maternal protein restriction in rats), in relation to birth weight and postnatal growth, blood pressure and cardiac structure and function. In doing so, we highlight the complexity of developmental programming, with regards to timing, degree of severity of the insult, genotype and the subsequent postnatal phenotype.

## 1. Introduction

The importance of maternal nutrition to growth of the foetus has long been recognised with inadequate maternal nutrition, as a result of undernutrition and/or malnutrition, linked to induction of intrauterine growth restriction (IUGR) and potential adverse impacts on lifelong health of the offspring. Importantly, over recent decades both epidemiological and experimental studies have shown an association between IUGR and an increased risk of cardiovascular disease later in life [1,2,3,4,5,6]. This association has been linked to “developmental programming” whereby sub-optimal growth during pregnancy results in fetal adaptations, including altered organogenesis, which can then render the offspring vulnerable to disease processes later in life [7]. The purpose of this review is to provide an overview of the current knowledge relating to IUGR and the long-term effects of IUGR on the heart. There is particular emphasis on maternal protein restriction which is a popular animal model used to induce IUGR and the subsequent effects on long-term cardiac health.

## 2. Low Birth Weight is Linked to Long-Term Cardiovascular Disease

It is now well established that events occurring during early life can also impact on long term levels of blood pressure and cardiovascular health [8,9,10,11], with impaired growth in early life leading to long term vulnerability to cardiovascular disease. Over recent decades many epidemiological studies have linked low birth weight with long term heart disease [12,13,14,15,16,17] and with other disease processes that are directly associated with an increased propensity for cardiovascular disease, such as metabolic disease [18,19], insulin resistance [20,21], non-insulin dependent diabetes [22,23], renal disease [24] and hypertension [25,26].

In 1977, Forsdahl was the first to report a close correlation between increased rates of death from ischaemic heart disease and poverty in childhood and adolescent years in Norway [27]. Similar relationships were reported in early studies from England and Wales [28]. In the 1980s Barker and colleagues reported in a cohort of 10,141 men from Hertfordshire, England, born between 1911 and 1930 that the incidence of death from ischaemic heart disease was highest in the men with lowest birth weights and weights at one year of age, compared to individuals of normal birth weight; this was independent of lifestyle factors [29]. This is when the importance of maternal diet during pregnancy to the long-term health of her offspring was first recognised.

Since then, there have been many epidemiological studies in many populations worldwide that have confirmed these observations [3,11,15,30,31,32,33,34,35]. Of particular interest are the findings of the Nurses’ Health Study in which the health of 121,700 women in the USA was retrospectively followed up from 1976 and interestingly, the strong associations between low birth weight and coronary heart disease remained after adjustments were made for adult smoking, physical activity, dietary habits and socio-economic status [3]. This association is strongest when there is accelerated body growth after birth [4,15,36,37,38,39,40] and collectively, these studies suggest that it is the accelerated postnatal growth that characteristically occurs in small-for-gestational age infants, rather than low birth weight per se, that leads to the increased risk of cardiovascular disease later in life.

Negative correlations between birth weight and levels of blood pressure in adult life are also now well established [41,42,43,44]. The first studies indicating that high blood pressure might have its origins in utero were population-based studies in the UK and other parts of Europe [8,9,31,45,46,47]. These studies have pointed out that an increase in birth weight was associated with a fall in blood pressure in adulthood. These correlations are reported to remain regardless of common risk factors such as alcohol consumption and body mass index in adulthood [48,49,50]. The results from a longitudinal study by Uiterwaal and colleagues following up 252 males and 231 females for 14 years demonstrated a strong and consistent inverse association between birth weight and systolic blood pressure after adjustment for body weight and height. This association persisted from adolescence into adulthood [49]. The links between IUGR and increased risk of disease in adulthood appear to be strongest when there is an accelerated postnatal catch up in growth [51,52].

## 3. Catch-Up Growth

The “catch-up” growth or “postnatal accelerated growth” hypothesis was proposed approximately fifteen years ago by Alan Lucas and Atul Singhal [53,54]. This hypothesis proposes deleterious consequences to offspring when postnatal growth rate exceeds otherwise normal linear growth, predisposing subjects to increased risk of developing metabolic and cardiovascular disease.

It is well established that postnatal weight gain is an important indicator for the programming of adult disease [55]. Accelerated weight gain in childhood is itself a risk factor for elevated blood pressure later in life [56,57] and this is likely to be compounded by low birth weight. Findings from a number of clinical studies have revealed that postnatal catch-up in growth in low birth weight subjects can lead to adverse effects on cognitive function [58], blood pressure [37], cardiac function [59,60,61], insulin sensitivity and secretion [62], development of type 2 diabetes [63] and obesity [64] both in childhood and in early adulthood. For example, in a study from Helsinki, low birth weight children who had not only caught-up in body weight with their age matched counterparts, but were heavier by the seventh year of life were shown to develop hypertension in adulthood [65] and coronary heart disease [15]. In another study metabolic syndrome has also been reported in men at 58 years of age [66] who were born of low birth weight and experienced accelerated catch-up growth in early adulthood, up to 18 years of age. In a prospective Australian study, where a longitudinal pregnancy cohort was followed up from birth until 13 years of age, it was reported that growth trajectory in childhood predicted cardiovascular risk; cardiovascular risk was high in adolescents with restricted prenatal growth followed by accelerated postnatal growth [38]. In addition, a prospective US study where data were collected from a large biracial cohort of pregnant women and their offspring concluded that increasing growth percentiles during any period of early childhood increases the risk for high blood pressure [37].

## 4. Low Birth Weight and IUGR

Low birth weight is defined as birth weight below 2.5 kg [67], irrespective of gestational age and is universal to all ethnic groups/populations (according to the World Health Organisation) and can result from inappropriate growth in utero [68], preterm birth [69] or a combination of both. This review focuses on the effect of IUGR rather than preterm birth.

In clinical practice IUGR is generally assigned to small for gestational age infants with a birth weight and/or birth length below the 10th percentile for gestational age [70]. It occurs as an abnormal restriction of foetal growth due to adverse genetic or environmental influences [71].

In general, growth restriction commencing from early pregnancy leads to proportional or symmetrical growth restriction, whereas in infants where there is mid-trimester or third trimester growth restriction there is disproportionate or asymmetrical growth restriction [72]. When there is symmetrical growth restriction the growth of the head, femur and abdomen is equally affected [71], whereas in asymmetric growth restriction there is disproportional growth of the foetus, with preferential blood flow to the brain, termed brain sparing, resulting in a baby with a relatively normal head size but a below normal body size [73]. This asymmetric type of growth restriction develops when oxygen or substrate supply to the foetus is reduced during the last trimester of pregnancy, often due to a reduced functional capacity of the placenta [74]. There are a number of studies suggesting that asymmetrical growth restriction in foetuses results in a worse outcome later in life than symmetrical growth restriction [75].

IUGR is not a specific disease per se but a manifestation of many maternal and foetal factors leading to poor foetal growth. There are many causes of IUGR including environmental and genetic factors. In general, IUGR usually results from nutrient and/or oxygen deprivation to the foetus, often due to both maternal and foetal factors [76]. Experimental evidence indicates that the primary environmental factor that regulates foetal growth in animals and humans is nutrient delivery to the foetus [70,77]. Nutrient delivery is dependent on maternal nutritional intake and adequate maternal blood flow, which is essential for normal placental function [78]. In developed countries placental insufficiency is the leading cause of IUGR [76], whereas in developing countries maternal malnutrition is the major cause of IUGR resulting from long-term nutrient deprivation to the growing foetus [79]. As a result of the early observations of the link between IUGR and long term disease the developmental programming hypothesis evolved.

## 5. Early Life Programming for Long-Term Disease

The concept that there are embryonic and foetal adaptive responses to a suboptimal intrauterine environment that result in permanent adverse consequences is consistent with the definition of “programming” [80,81]. “Programming” refers to the idea that an insult or stimulus applied during a critical or sensitive period of development can have long lasting or persistent effects on the structure or function of an organism [53]; the “programming” can be either beneficial or detrimental to long term health. Both prenatal life and early postnatal life are “critical periods” that are characterised by a high degree of plasticity [82,83,84] and a high cell proliferation rate in the developing tissues [85,86]. Therefore, exposure to an adverse stimulus during these “critical periods” can lead to detrimental consequences in the growth of tissues and organs [55,87], which in turn, can cause persistent alterations in body function. In addition, adaptive programming of the foetus to IUGR can lead to modifications of biochemical and hormonal pathways within the foetus, again rendering the individual susceptible to disease later in life [88].

Potential programming effects on tissue structure and on the number of functional units formed in vital organs.

**Figure 1:**
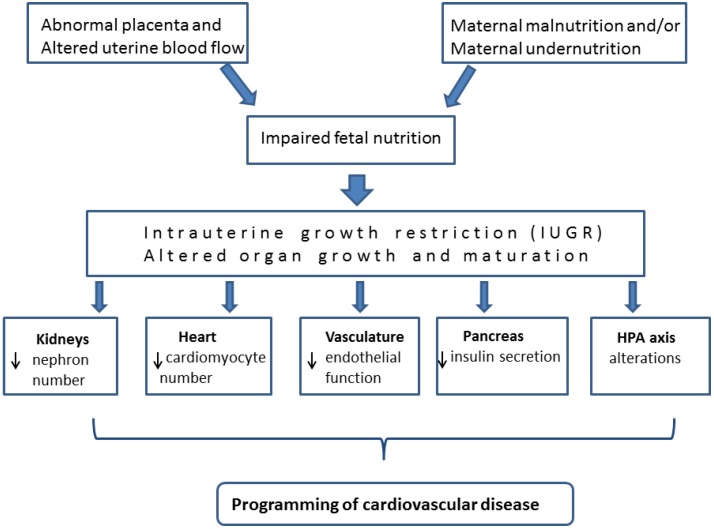
Diagram showing how impaired maternal nutrition and/or abnormal placental function leads to intrauterine growth restriction (IUGR) and subsequent changes to organs that play a key role in cardiovascular function. These changes have the potential to program for long-term cardiovascular disease.

Adverse environmental factors acting during the developmental period have the potential to disturb the processes of cell proliferation and differentiation [89]. The vulnerability of particular organs and organ systems to exposure to insults during gestation usually coincides with the periods in development when the organs are first forming and/or during “critical periods” of cellular proliferation and differentiation [85,90]. Indeed, a reduction in cell number, or a change in the balance of cell types within tissues, has been observed in a number of animal models in response to an altered intrauterine environment [91,92]. Such changes may account for subsequent alterations in gene expression and physiological function. Certainly, a reduction in the complement of the functional units within vital organs has the potential to adversely impact on the functional capacity and adaptive capabilities in adulthood. This is especially important given that the proliferative capacity of the functional units in many vital organs usually ceases prior to birth, or soon after birth, hence reduced foetal growth can lead to a lifelong deficit in the functional capacity of vital organs (Figure 1).

For example, a reduction in:

(1) Nephron number has been observed in offspring in response to a maternal prenatal low-protein diet in the rat [93,94], mouse [95], and following uterine artery ligation in the guinea pig [96] and rabbit [97] and following placental embolization in sheep [98].
(2) Total cardiomyocyte number in the offspring of rats exposed to maternal protein restriction or placental insufficiency during pregnancy [99] and in lambs exposed to placental insufficiency the total complement of cardiomyocytes has been shown to be directly related to heart size [100].
(3) The numbers of secondary muscle fibres has been reported in the young offspring of a variety of species, including rats [101], pigs [102] and sheep [103] following maternal undernutrition during the critical proliferative period for muscle fibre development.
(4) Total pancreatic weight, islet cell mass and the relative proportion of β-cells within the islets [104] has been reported to be lower in IUGR rat offspring.

Furthermore, it has been shown that the vasculature undergoes permanent changes in reactivity as a result of maternal nutrient restriction [105]. In addition, there are reports of persistent alterations of the hypothalamic-pituitary-adrenal (HPA) axis of IUGR rat offspring [106,107] and this is postulated to play a critical role in the observed association between foetal growth restriction and subsequent cardiovascular and metabolic diseases.

Figure 1 collectively shows the sequence of events that can potentially lead to the programming for increased risk of cardiovascular disease in IUGR offspring.

## 6. Animal Model of IUGR—Maternal Protein Restriction in Rats

Much of our knowledge relating to the short term and long-term effects of IUGR has been derived from animal studies. A number of animal models of poor maternal nutrition and/or placental insufficiency have been developed over recent years to investigate the causes and consequences of IUGR. A variety of species have been studied, including: rodents, sheep and primates; and both, maternal dietary manipulations or surgical interventional techniques have been employed [108,109,110,111,112,113,114]. One of the most extensively studied and well-characterised animal models is maternal protein restriction in rats. Regardless of how severe the protein restriction is (mild- 9% diet or severe- 5% diet) the end result is reduction in body weight of the offspring [115,116,117,118,119].

In our laboratory over the past decade we have comprehensively examined the cardiovascular phenotype of rat offspring following maternal protein restriction. However, as our studies have progressed, it has become clearly apparent, that the cardiovascular and metabolic phenotype of the offspring using this model differs between different laboratories, which likely relates to subtle differences in study design. This in turn, makes comparison of the findings between studies difficult. For example, there are differences in the strain of rats studied, levels of maternal protein restriction in the diet, timing of administration of the diet to the dams and postnatal differences in body growth and levels of blood pressure of the offspring. These differences are highlighted in Table 1.

In our studies, Wistar-Kyoto (WKY) female breeder rats are fed a low protein diet for two weeks prior to birth to get the dams accustomed to the diet, then during pregnancy and for two weeks during lactation as the rodent organ systems are still developing in the early postnatal period. To avoid a high mortality rate in the offspring [120] we have chosen moderate protein restriction (8.7% casein in the diet) for the dams [121,122,123,124,125,126,127,128], rather than a more severe protein deprivation (6% casein) that is sometimes used by other investigators [129,130,131].

In the following sections we compare our findings with others; in doing so, we highlight differences in the maternal protein restriction model, which may account for conflicting findings between laboratories (Table 1).

**Table 1:** Studies investigating the effects of administration of a maternal low protein diet (LPD) in rats on the phenotype of the offspring—highlighting differences in the rat strains studied, severity of the dietary protein restriction and the timing of the diet administration. The table is organized according to the severity of maternal protein restriction. Studies conducted in our laboratory are highlighted in grey.

Author and Year of the Study	Rat Strain and Age of Offspring at Investigation	Diets	Timing of the Diet	Major Findings in the LPD Group
Woods* et al.* 2004 [93]	Sprague-Dawley 22 weeks	NPD: 19.0% casein LPD: 5.0% casein	During pregnancy	↓ birth wt ↓ body wt at 22 weeks ↓ nephron number ↑ MAP at 22 weeks
Dagan* et al.* 2009 [132]	Sprague-Dawley 6 weeks	NPD: 20.0% casein LPD: 6.0% casein	During pregnancy	↑ SBP at 6 weeks birth wt and body wt were not reported
Habib* et al.* 2011 [133]	Sprague-Dawley 9 and 12 weeks	NPD: 20.0% casein LPD: 6.0% casein	During pregnancy	↓ birth wt ↓ body wt at 12 weeks ↑SBP at 9 and 12 weeks ↓ nephron number
Langley and Jackson, 1994 [134]	Wistar 9 and 21 weeks	NPD: 18.0% casein LPD: 12.0% , 9%, and 6% casein	2 weeks prior to and during pregnancy	↓ body wt in 6%LPD group at 21 weeks ↔ body wt in 9% and 12% LPD group at 21 weeks ↑ SBP in all three LPD groups at 9 weeks ↑ SBP in 9% and 6% LPD groups at 21 weeks
Manning* et al.* 2002 [130]	Sprague-Dawley 4 and 8 weeks	NPD: 20.0% casein LPD: 6.0% casein	During pregnancy	↓ birth wt ↔ body wt at 4 weeks ↔ SBP at 4 weeks ↑ SBP at 8 weeks
Manning and Vehaskari, 2001 [135]	Sprague-Dawley 4, 8 and 45 weeks	NPD: 20.0% casein LPD: 6.0% casein	During pregnancy	↓birth wt ↔ body wt at 4 weeks ↑ SBP at 8 until 40 weeks ↓ survival rate at 45 weeks
Sathishkumar* et al.* 2009 [131]	Sprague-Dawley 52 weeks	NPD: 18.0% casein LPD: 6.0% casein	During pregnancy	↓ birth wt ↓ body weight at 52 weeks ↑ MAP at 52 weeks ↑ vascular contraction ↓ vascular relaxation
Tonkiss* et al.* 1998 [136]	Sprague-Dawley 14 weeks	NPD: 25.0% casein LPD: 6.0% casein	5 weeks prior to and during pregnancy	↓ birth wt ↔ body wt at 14 weeks ↑ DBP at 14 weeks ↔ SBP at 14 weeks
Vehaskari* et al.* 2001 [137]	Sprague-Dawley 8 weeks and 78 weeks	NPD: 20.0% casein LPD: 6.0% casein	During pregnancy	↓birth wt ↔ body wt at 2 weeks ↔ SBP at 4 weeks ↑ SBP at 8 weeks ↓ survival at 78 weeks
Coupe* et al.* 2009 [138]	Sprague-Dawley 36 weeks	NPD: 20.0% casein LPD: 8.0% casein	During pregnancy	↓ birth wt ↔ body wt at 36 weeks ↓ triglycerides, glucose
Hoppe* et al.* 2007 [95]	Sprague-Dawley 4 and 19 weeks	NPD: 20.0% casein LPD: 8.0% casein	2 weeks prior to and during pregnancy and 3 weeks postnatally	↓ body wt at 4 and 19 weeks ↓ organ weights (except brain) ↓ MAP at 19 weeks ↓ nephron number
Ozanne* et al.* 1996 [121]	Wistar 12 weeks	NPD: 20.0% casein LPD: 8.0% casein	During pregnancy and 3weeks postnatally	↓ body wt at 12 weeks ↓ size of tibialis anterior muscle ↑ insulin sensitivity
Plank* et al.* 2006 [124]	Wistar 10 weeks	NPD: 17.0% casein LPD: 8.0% casein	During pregnancy	↓ birth wt ↓ body length at birth ↔ body wt at 8 weeks ↔ MAP at 10 weeks ↑ inflammatory markers
Plank* et al.* 2008 [139]	Wistar 17 weeks	NPD: 17.0% casein LPD: 8.0% casein	During pregnancy	↓ birth wt ↓ body length at birth ↓ body wt at 3weeks ↔ body length at 3 weeks ↔ body wt at 17 weeks ↑ MAP at 17 weeks
Zeng* et al.* 2013 [140]	Sprague-Dawley 1 to 78 weeks	NPD: 20.0% casein LPD: 8.0% casein	During pregnancy	↓ birth weight ↔ body wt at 4 and 8 weeks ↑ body wt at 12, 52 and 78 weeks ↑ insulin secretion
Menendez-Castro *et al.* 2011 [141]	Wistar 10 weeks	NPD: 17.2% casein LPD: 8.4% casein	During pregnancy	↓ birth wt ↓ body wt at 10 weeks ↔ relative heart wt ↔ MAP at 10 weeks ↔ SBP at 10 weeks ↑ myocardial collagen I and collagen IV at 10 weeks
Menendez-Castro *et al.* 2014 [6]	Wistar 10 weeks	NPD: 17.2% casein LPD: 8.4% casein	During pregnancy	↓ ejection fraction ↓ fractional shortening ↑ LV diameters at systole and diastole at 10 weeks
Woods* et al.* 2001 [122]	Sprague-Dawley 21 weeks	NPD: 19.0% casein LPD: 8.5% casein	During pregnancy	↓ birth wt ↔ body wt at 3 and 21 weeks ↓ nephron number at 21 weeks ↑ MAP at 21 weeks
Woods* et al.* 2005 [142]	Sprague-Dawley 22 and 50 weeks	NPD: 19.0% casein LPD: 8.5% casein	During pregnancy	↓ birth wt ↔ body wt at 4 and 22 weeks ↓ body wt at 50 weeks ↔ MAP at 22 and 50 weeks ↔ nephron number
Corstius* et al.* 2005 [99]	WKY at birth	NPD: 20.0% casein LPD: 8.7% casein	2 weeks prior to and during pregnancy and 2 weeks postnatally	↓ birth wt ↓ heart wt ↓ cardiomyocyte number
Lim* et al.* 2006 [143]	WKY 24 weeks	NPD: 20.0% casein LPD: 8.7% casein	2 weeks prior to and during pregnancy and 2 weeks postnatally	↓ body wt at 2 and 24 weeks ↔ heart wt at 24 weeks ↑ LV + S interstitial fibrosis
Lim* et al.* 2010 [144]	WKY 4 weeks	NPD: 20.0% casein LPD: 8.7% casein	2 weeks prior to and during pregnancy and 2 weeks postnatally	↓ body wt at 4 weeks ↑ relative heart volume at 4weeks ↔ cardiomyocyte number
Lim* et al.* 2011a [145]	WKY 32 weeks	NPD: 20.0% casein LPD: 8.7% casein	2 weeks prior to and during pregnancy and 2 weeks postnatally	↓ birth wt ↓ body wt at 32 weeks ↑ insulin sensitivity ↔ SBP at 32 weeks ↔ body composition ↔ locomotor activity
Lim* et al.* 2011b [146]	WKY 32 weeks	NPD: 20.0% casein LPD: 8.7% casein	2 weeks prior to and during pregnancy and 2 weeks postnatally	↓ birth wt ↓ body wt at 32 weeks ↓ kidney wt at 32 weeks ↔ MAP at 32 weeks
Lim* et al.* 2012 [147]	WKY 32 weeks	NPD: 20.0% casein LPD: 8.7% casein	2 weeks prior to and during pregnancy and 2 weeks postnatally	↓ body wt at 32 ↑ relative heart wt ↑ LV + S interstitial fibrosis ↔ SBP at 32 weeks
Zimanyi* et al.* 2004 [117]	WKY 4 and 40 weeks	NPD: 20.0% casein LPD: 8.7% casein	2 weeks prior to and during pregnancy and 2 weeks postnatally	↓ birth wt ↓ body wt at 40 weeks ↓ kidney volume, nephron number at 4 weeks ↔ SBP at 40 weeks
Zimanyi* et al.* 2006 [94]	WKY 4 and 24 weeks	NPD: 20.0% casein LPD: 8.7% casein	2 weeks prior to and during pregnancy and 2 weeks postnatally	↓ birth wt ↓ body wt at 24 weeks ↓ nephron number at 4 weeks ↔ MAP at 24 weeks
Zohdi* et al.* 2011 [148]	WKY 14 weeks	NPD: 20.0% casein LPD: 8.7% casein	2 weeks prior to and during pregnancy and 2 weeks postnatally	↓ birth wt ↓ body wt at 14 weeks ↓ heart wt at 14 weeks ↔ MAP at 14 weeks ↑ arterial elastance, total peripheral resistance
Zohdi* et al.* 2013 [149]	WKY 18 weeks	NPD: 20.0% casein LPD: 8.7% casein	2 weeks prior to and during pregnancy and 2 weeks postnatally	↓ birth wt ↓ body wt at 18 weeks ↔ heart weight at 18 weeks ↔ cardiac fibrosis ↑ biochemical composition in the heart
Zohdi* et al.* 2014 [150]	WKY 18 weeks	NPD: 20.0% casein LPD: 8.7% casein	2 weeks prior to and during pregnancy and 2 weeks postnatally	↓ birth wt ↓ body wt at 18 weeks ↓ aortic peak systolic velocity at 18 weeks ↔ SBP at 18 weeks ↔ basal cardiac function at 18 weeks
Alwasel and Ashton, 2009 [125]	Wistar 4 weeks	NPD: 18.0% casein LPD: 9.0% casein	During pregnancy	↔ body wt at 4 weeks ↑ MAP
Bellinger* et al.* 2006 [123]	Wistar 36 weeks and 72 weeks	NPD: 18.0% casein LPD: 9.0% casein	During pregnancy	↔ birth wt ↔ body wt at 36 weeks ↓ body wt at 72 weeks
Boubred* et al.* 2009 [126]	Sprague-Dawley 4, 8, 16, 52 and 100 weeks	NPD: 22.0% casein LPD: 9.0% casein	During pregnancy	↓ birth wt ↔ SBP at 4 and 52 weeks ↑ SBP at 8 and 16 weeks ↔ body wt at 100 weeks
Brawley* et al.* 2003 [151]	Wistar 18 weeks	NPD: 18.0% casein LPD: 9.0% casein	During pregnancy	↔ birth wt ↓ body wt at 9 weeks ↔ body wt at 18 weeks ↑ SBP at 18 weeks
Cheema* et al.* 2005 [118]	Wistar 1–40 weeks	NPD: 18.0% casein LPD: 9.0% casein	2 weeks prior and during pregnancy	↓ birth wt ↓ body wt at 40 weeks LV hypertrophy at 40 weeks
Elmes* et al.* 2007 [152]	Wistar 4, 8 and 24 weeks	NPD: 18.0% casein LPD: 9.0% casein	During pregnancy	↑ SBP at 4 and 8 weeks ↔ baseline cardiac function ↓ recovery to myocardial ischemia at 24 weeks
Gardner* et al.* 1997 [153]	Wistar 6 weeks	NPD: 18.0% casein LPD: 9.0% casein	2 weeks prior to and during pregnancy	↔ body wt at 6 weeks ↑ SBP at 6 weeks
Harrison and Langley-Evans, 2009 [154]	Wistar, 8 and 10 weeks F_1_, F_2_, F_3_ generational study	NPD: 18.0% casein LPD: 9.0% casein	During pregnancy	↔ body wt at 10 weeks in all generations ↑ SBP at 8 weeks in F_1_ and F_2_↓ nephron number in F_1_ and F_2_
Langley-Evans* et al.* 1994 [155]	Wistar 4 weeks	NPD: 18.0% casein LPD: 9.0% casein	2 weeks prior to and during pregnancy	↓ birth wt ↑ SBP at 4 weeks
Langley-Evans* et al.* 1996 [156]	Wistar 7 weeks	NPD: 18.0% casein LPD: 9.0% casein	During pregnancy	↓ birth wt ↔ body wt at 7 weeks ↑ SBP at 7 weeks
Langley-Evans* et al.* 1999 [116]	Wistar 4 and 19 weeks	NPD: 18.0% casein LPD: 9.0% casein	2 weeks prior to and during pregnancy	↔ body wt at 4 and 19 weeks ↑ SBP at 4 and 19 weeks
McMullen and Langley-Evans, 2005 [157]	Wistar 4 weeks	NPD: 18.0% casein LPD: 9.0% casein	During pregnancy	↔ birth wt ↔ body wt at 4 weeks ↑ SBP at 4 weeks
McMullen* et al.* 2004 [158]	Wistar 4 weeks	NPD: 18.0% casein LPD: 9.0% casein	2 weeks prior to and during pregnancy	↑ SBP at 4 weeks ↓ nephron number birth wt and body wt were not reported
Mehta* et al.* 2002 [159]	Wistar 52 weeks	NPD: 18.0% casein LPD: 9.0% casein	During pregnancy	↔ birth wt ↔ body wt at 52 weeks ↓ bone mineral content ↓ bone mineral density
Nwagwu* et al.* 2000 [160]	Wistar 4, 12 and 20 weeks	NPD: 18.0% casein LPD: 9.0% casein	During pregnancy	↔ birth wt ↑ SBP at all three ages ↓ kidney: body wt at 4weeks ↔ kidney morphometry at 12 weeks
Pladys* et al.* 2005 [161]	Wistar 9 to12 weeks	NPD: 18.0% casein LPD: 9.0% casein	During pregnancy	↔ birth wt ↔ body wt at 12 weeks ↑ MAP at 9 to 12 weeks ↔ adult arterial structure
Sherman and Langley-Evans, 2000 [127]	Wistar 4 and 12 weeks	NPD: 18.0% casein LPD: 9.0% casein	During pregnancy	↑ body wt at 4 weeks ↔ body wt at 12 weeks ↑ SBP at 4 and 12 weeks
Swali* et al.* 2010 [128]	Wistar 4, 8 and 12 weeks	NPD: 18.0% casein LPD: 9.0% casein	During pregnancy	↔ birth wt ↔ body wt between 4 and 12 weeks ↔ SBP at 4 weeks ↑ SBP at 8 weeks ↓ SBP at 12 weeks
Tappia* et al.* 2005 [162]	Wistar 3 weeks	NPD: 18.0% casein LPD: 9.0% casein	2 weeks prior to and during pregnancy	↓ birth wt ↓ saturated cardiac fatty acids ↑ unsaturated cardiac fatty acids
Tappia* et al.* 2011 [163]	Wistar 1, 4 and 8 weeks	NPD: 18.0% casein LPD: 9.0% casein	2 weeks prior to and during pregnancy	↑ LV internal diameter at all ages ↑ LV wall thickness at 4 weeks
Torrens* et al.* 2006 [164]	Wistar 15 weeks	NPD: 18.0% casein LPD: 9.0% casein	2 weeks prior to and during pregnancy	↔ birth weights ↑ SBP at 15 weeks
Ohishi* et al.* 2012 [165]	Sprague-Dawley 10 weeks	NPD: 20.0% casein LPD: 10.0% casein	During pregnancy and 3 weeks postnatally	↓ body wt at 10 weeks ↓ grip strength at 10 weeks ↑ motor activity at 10 weeks
Reyes-Castro* et al.* 2011 [166]	Wistar 17 and 21 weeks	NPD: 20.0% casein LPD: 10.0% casein	During pregnancy and 3 weeks postnatally	↓ birth wt ↔ body length at birth ↓ body wt at 17 and 21 weeks ↓ cognitive function at 21 weeks
Zambrano* et al.* 2006 [167]	Wistar 16 and 18 weeks	NPD: 20.0% casein LPD: 10.0% casein	During pregnancy and 3 weeks postnatally	↓ birth wt in females ↔ birth wt in males ↓ body wt at 3 and 18 weeks for both sexes ↑ insulin sensitivity

MAP: mean arterial pressure; NPD: normal protein diet; LV: left ventricle; LV + S: left ventricle plus interventricular septum; SBP: systolic blood pressure; wt: weight; WKY: Wistar-Kyoto; ↑: increased; ↓: decreased; ↔: unchanged.

## 7. Low Birth Weight and Postnatal Body Growth

In accordance with our findings, many laboratories worldwide have shown that administration of a protein restricted diet to rat dams during pregnancy leads to growth restriction in the offspring (Table 1). Given the relative consistency of these findings, maternal protein restriction in rats has now become one of the most commonly used animal models of IUGR (Table 1). To the contrary, however, the long-term effects on body weight are not always the same; in some laboratories the offspring undergo postnatal catch up in body growth, whereas in others the body weight of the offspring remains attenuated throughout life (Table 1). It is a consistent finding in our laboratory that the low protein diet (LPD) offspring are born small and they then remain significantly smaller throughout life when compared to normal protein diet (NPD) control offspring [99,117,143,144,145,147,148,149]; this is also reported in some other research groups [95,141]. In contrast, catch-up in body growth in the IUGR rat offspring is often reported following maternal protein restriction [51,137,140].

Why there are differences in postnatal growth in the offspring between studies is unknown. It is conceivable that it may be the prolonged maternal protein restriction after birth for two weeks during lactation in our studies that leads to the persistent attenuation of body growth in the LPD offspring. In our studies, we have chosen to feed the rat dams the specialized diets (LPD and NPD) for the first two weeks of lactation as rats are an altricial species and are born at a time when their organs are very immature. For instance, in the heart maturation of cardiomyocytes, which occurs late in gestation in humans, occurs in the first two weeks after birth in the rat [168]. Likewise, in the kidney nephrogenesis is complete by term birth in the human but continues in the first two weeks after birth in the rat. Hence, in order to more closely mimic the effect of IUGR on organ development, we have considered it appropriate to continue the maternal protein restriction until two weeks after birth.

In addition, there is another important difference in relation to the dietary feeding regime to the rat dams in our studies, compared to many other studies. In our studies, the dams commence the diet 2 weeks prior to mating, in order to get the dams accustomed to the specialised diets. In contrast, in the majority of studies utilising the maternal protein restriction model, they have commenced feeding the diet to the dams at the beginning of pregnancy. Hence, it may be the feeding of the low protein diet to the dams during the periconceptional period that has led to the long-term attenuation of postnatal growth and to some of the other differences in findings, compared to other studies using the maternal protein restriction model. Indeed, there have been a number of recent studies demonstrating long-term effects on the offspring, as a result of insults; including impaired nutrition, experienced by the mothers at around the time of conception [92,169,170,171,172], thus highlighting the importance of the periconceptional period in long-term programming [129,173,174]. Hence, in future studies, it would certainly be beneficial to explore the effects of the timing of the low protein diet to the dams on the long-term outcomes in the offspring, in order to differentiate the importance of the periconceptional, pregnancy and lactation periods in mediating the long-term effects.

## 8. Elevated Blood Pressure in Adulthood is not a Direct Corollary of IUGR

Over recent decades there have been many epidemiological studies that describe a direct link between being born small with an elevation of blood pressure in adulthood [8,31,48,175,176,177]. Interestingly, however, our studies using the maternal protein restriction model in rats do not support this concept. The blood pressure measurements in our studies have been performed using various methods; (tail cuff plethysmography, intra-arterial and high fidelity pressure sensor; and the outcome is always the same. The IUGR LPD offspring remain normotensive through to adulthood and their levels of blood pressure are not different to the non-IUGR NPD offspring, thus demonstrating that an elevation in blood pressure is not a direct corollary of IUGR.

The findings in relation to the effects of IUGR on blood pressure later in life using animal models differs widely amongst studies; some studies report no effects on blood pressure, whereas others report an elevation in blood pressure in adulthood (Table 1). In our studies the absence of an elevation in blood pressure in the adult IUGR offspring is in agreement with other previously reported studies [95,142,178] yet contrary to others [93,116,132,133,134,139,151,155,156]. Collectively, the findings clearly indicate that induction of hypertension in adulthood is not a direct corollary of being born small. The question thus arises why do the IUGR offspring in some studies develop high blood pressure in adulthood and in other studies blood pressure in adult IUGR offspring is not affected? There are a number of potential explanations for the discrepancies in findings. It may be that when body growth remains attenuated throughout life that blood pressure is not affected. It is likely that the cardiovascular system is programmed in utero and hence, cardiovascular function may only be adversely affected when there is a mismatch in prenatal and postnatal growth. Certainly, there are a number of experimental studies supporting this concept in the programming of metabolic disease. Importantly in this regard, using our model of maternal protein restriction in rats there is improved insulin sensitivity supporting the concept that it is the “mismatch” in prenatal and postnatal growth that leads to impaired glucose metabolism [179,180,181,182,183], the majority of studies shown in Table 1 support this concept in relation to the programming of hypertension [122,126,130,135,156]. Hence, when the IUGR LPD offspring experience accelerated postnatal growth, such that body weight is no longer different to the non-IUGR NPD offspring, these offspring generally exhibit a significant elevation in blood pressure. However, it is important to point out, that there are a few studies shown in Table 1, where body weights in the LPD offspring, at the time of examination, were still less than controls but blood pressure was significantly elevated [93,131,133].

An alternative explanation for the differences observed in levels of adult blood pressure may relate to whether the protocols used to measure blood pressure leads to stress in the animals. Indeed, there have been a number of studies suggesting that it is an elevated stress response in IUGR offspring that subsequently leads to the elevation in blood pressure rather than a direct etiological effect [39,128,136,178,184,185]. Hence, when rats are stressed during some procedures used to measure blood pressure, there will be an elevated stress response in the IUGR offspring, and hence, a concomitant elevated blood pressure response. This is similar to the “white coat hypertension” often experienced by human subjects when their blood pressure is measured in a clinical setting (and they have become stressed during the procedure) [186,187,188,189,190]. Certainly measurements of tail-cuff blood pressure can lead to stress in the animals, especially if they have not been well conditioned to the procedure. In early studies in the field, “one off” measurements were performed using tail-cuffs [155,156] and it is highly likely, that the rats were stressed during these procedures. In such studies, blood pressure would have been elevated in both IUGR and non-IUGR offspring during these procedures; however, if there was an elevated stress response in IUGR offspring this would lead to a greater elevation of blood pressure in these offspring. Van Abeelen and colleagues addressed this issue in a recent systemic review where they included 101 experimental studies from sheep, guinea pigs, rats and mice looking at the effects of maternal undernutrition (34 studies of maternal general undernutrition and 67 studies of low protein undernutrition) on the blood pressure in the offspring [185]. They pointed out that the values of blood pressure reported from tail-cuff measurements overestimate the “true value” of blood pressure when taken by a direct method using intra-arterial catheters. Furthermore, they have indicated that a direct comparison between tail-cuff and radiotelemetry would be beneficial when conducting studies [185]. In this regard, Swali and colleagues have reported simultaneous measurements of blood pressure, using tail-cuffs and telemetry, in IUGR and control offspring during baseline and under various stress conditions [128]. They found a good correlation between tail-cuff and radiotelemetry derived blood pressure data in control rats; however, in the IUGR group the tail-cuff method revealed hypertension at eight weeks of age but the telemetry method indicated significantly lower blood pressure at twelve weeks of age compared to controls [128]. Hence, their interpretation of these findings was that the increase in systolic blood pressure in LPD offspring reflects an increase in peripheral vascular resistance as well as change in the degree of amplification of blood pressure between central and peripheral regions.

## 9. Cardiac Remodelling in the Adult IUGR Heart with Normal Basal Function

In our analyses of the adult hearts of the IUGR LPD offspring we have no evidence of overt structural abnormalities in the myocardium of LPD offspring compared to NPD offspring in early adulthood (18 weeks of age) as assessed by echocardiography [150] and there is no significant difference in the amount of interstitial collagen deposition within the myocardium between the LPD and NPD groups [149]. Interestingly, however, when the biochemical composition of the left ventricle was assessed using FTIR micro-spectroscopy [149] there were marked differences detected in the biochemical spectra of the growth-restricted myocardium. In particular, there was a significant increase in the intensity of lipids, proteoglycans and carbohydrates as indicated by the increased absorbance of the 1455 and 1388 cm^−1^, 1228 cm^−1^, 1038 cm^−1^ bands, respectively. However, the protein, lipid and proteoglycan spatial distribution was similar within the myocardium of the left ventricular free wall and interventricular septum of the LPD and NPD adult offspring [149]. Interestingly, the spatial distribution of carbohydrates was different in the IUGR and non-IUGR hearts at 18 weeks of age with the most striking difference between the NPD and LPD myocardium observed in the absorbance band at 1228 cm^−1^, which is due to the presence of proteoglycans. Importantly, in this regard it has been shown that an increase in proteoglycan deposition can ultimately affect cardiac performance [191,192]. The increase in carbohydrate content in the myocardium of IUGR offspring may be indicative of altered glucose metabolism within the LPD offspring. Certainly, experimental studies link IUGR with programming of altered glucose metabolism [193,194]. We have not directly assessed glucose metabolism in the IUGR offspring in our model of maternal protein restriction. However, in a previous study in our laboratory we have shown that maternal protein restriction leads to the programming of improved postnatal whole body insulin sensitivity when postnatal growth is similar to that in utero [145], which does not support the concept that glucose metabolism is impaired. Future studies are required to further investigate the cause of the increased carbohydrate content in the LV myocardium and determine whether it relates to altered glucose metabolism. It is conceivable that the biochemical changes that we have observed in the heart of the adult IUGR LPD offspring may have developed during foetal life. In support of our findings, Tappia and colleagues showed an altered phospholipid profile and fatty acid content in IUGR offspring at birth [162].

Although no differences in myocardial collagen were observed between LPD and NPD offspring at 18 weeks of age we have detected an increase in interstitial fibrosis in LPD offspring at 24 weeks of age [143] and at 32 weeks of age [147]; hence, it is conceivable that there may be an exacerbated deposition of collagen within the myocardium as the LPD offspring age. Interestingly, at 18 weeks of age we found minimal evidence of overt cardiac dysfunction under basal conditions in the IUGR offspring as assessed using both echocardiography and P-V catheterization techniques; fractional shortening a measure of myocardial contractility was normal [150]. Likewise, in another study from our laboratory there was preserved fractional shortening of the cardiac muscle in the IUGR offspring at 32 weeks of age [147]. Given our findings in relation to blood pressure and body weight, it is not really surprising that basal cardiac function was normal in the IUGR offspring; with normal blood pressure and attenuated postnatal body growth of the IUGR offspring the hemodynamic demands on the cardiovascular system are not likely to have increased in the IUGR offspring in adulthood. However, it is important to note, that when the hearts were challenged with dobutamine that the increase in both stroke volume and cardiac output were attenuated and the arterial elastance remained significantly elevated in the IUGR offspring, indicative of increased afterload on the heart [148]. In addition, echocardiographic analysis demonstrated a significant increase in end systolic dimensions and a significant reduction in aortic peak systolic velocity; which may indicate direct adverse effects on aortic compliance or mild impairment of systolic function. Others have shown in a rat model of maternal protein restriction that ejection fraction is significantly depressed in IUGR offspring very early in life at two weeks of age but subsequently normalised with no difference in ejection fraction between the IUGR and control offspring at 40 weeks of age as assessed by echocardiography [118]. Contrary to our findings, however, Menendez-Castro and colleagues have reported a significantly reduced ejection fraction as evaluated by echocardiography early in life at 10 weeks of age in IUGR offspring exposed to maternal protein restriction even though blood pressure was normal [6].

## 10. Challenging the Adaptive Capabilities of the IUGR Heart

Over recent years we have tested the hypothesis that IUGR acts as a primary insult to the heart, rendering the heart susceptible to secondary postnatal insults, such as hypertension, high salt diet and hyperglycaemia. It is well known that hypertension leads to left ventricular hypertrophy [195,196,197,198,199] and hence, it was considered likely that when the adaptive capabilities of the IUGR heart are challenged by hypertension, the pathological changes that ensue in the heart would be exacerbated in the heart of offspring that were born IUGR. In our studies, hypertension was induced by continuous infusion, at a pressor dose, of the potent vasopressor hormone angiotensin II (Ang II) [200]. Importantly, given that the IUGR LPD offspring in our studies do not normally develop high blood pressure in adulthood, we were able to look at the secondary effects of induction of hypertension, independent of an underlying primary hypertension.

Contrary to our initial hypothesis, when hypertension was induced as a secondary cardiac insult, the response to hypertension was not exacerbated in the IUGR offspring. The cardiac hypertrophic growth response to Ang II infusion, as assessed using echocardiography, was not different between the IUGR and non-IUGR offspring; however, there were differences in cardiac tissue structure. Unexpectedly, in the Ang II infused IUGR adult offspring the levels of interstitial collagen in the left ventricle myocardium was markedly reduced when compared to the non-IUGR offspring (unpublished observations from our laboratory). Hence, our findings do not support the concept that the IUGR heart is necessarily more vulnerable to hypertension in adulthood and importantly, our findings suggest that in some circumstances the IUGR heart may be somewhat protected from adverse remodelling. Further studies are required to elucidate the mechanisms for the reduced deposition of collagen.

In other studies in our laboratory we have examined the effects of induction of diabetes in adulthood, as a secondary postnatal insult, on the growth of the IUGR and non-IUGR heart [147]. Similar to that seen with induction of hypertension, the overall cardiac growth response to induction of diabetes, assessed by echocardiography, was not different between the IUGR and non-IUGR hearts. Importantly, however, the level of fibrosis was significantly greater in the IUGR diabetic hearts compared to non-IUGR diabetic hearts [147]. Collectively, our findings suggest that the IUGR heart may be better able to structurally adapt to a haemodynamic challenge, but not to the challenge of hyperglycaemia. However, this may be a somewhat simplistic interpretation of findings given that the mechanisms of the induction of cardiac hypertrophy are complex and both secondary insults are likely to involve haemodynamic and endocrine mechanisms.

There have been a number of studies that have examined the effect of a high salt diet administered postnatally, as a secondary lifestyle insult, on blood pressure in IUGR offspring [95,117,201,202,203]. Interestingly, several studies have shown that the increase in blood pressure in response to a high salt diet is similar between IUGR offspring and non-IUGR offspring [117,204], whereas some report salt-sensitive hypertension [93] and others report a reduction in blood pressure [95]. In our maternal protein restriction model it was previously shown that there was no difference in the elevation of blood pressure response to a high salt diet between LPD and NPD offspring [117]. Given that a high salt diet, is linked to induction of cardiac fibrosis [205,206] it would be interesting in future studies to compare the structural remodelling in the LPD IUGR offspring relative to NPD controls following the feeding of a high salt diet in adolescence/adulthood.

## 11. Conclusions

In summary, the findings of this review highlight the importance of maternal diet on the long-term cardiovascular outcomes of the offspring. Upon comparison of the findings between different laboratories using rat models of maternal protein restriction we highlight the many differences in the cardiovascular phenotype of the offspring between studies, which may relate to the rat strains studied, severity of the dietary protein restriction and the timing of the diet administration. In addition, this review emphasizes the complexity of the mechanisms relating to the developmental programming of heart disease and highlights directions for future research that are required to establish the importance of the periconceptional, pregnancy, lactational and post-weaning windows in life-long developmental programming.

## References

[1] Barker D.J., Gluckman P.D., Godfrey K.M., Harding J.E., Owens J.A., Robinson J.S. (1993). Fetal nutrition and cardiovascular disease in adult life. Lancet.

[2] Barker D.J. (1997). Intra-uterine programming of the adult cardiovascular system. Curr. Opin. Nephrol. Hypertens..

[3] Rich-Edwards J.W., Stampfer M.J., Manson J.E., Rosner B., Hankinson S.E., Colditz G.A., Willett W.C., Hennekens C.H. (1997). Birth weight and risk of cardiovascular disease in a cohort of women followed up since 1976. BMJ.

[4] Forsen T., Eriksson J.G., Tuomilehto J., Osmond C., Barker D.J. (1999). Growth in utero and during childhood among women who develop coronary heart disease: Longitudinal study. BMJ.

[5] Tappia P.S., Guzman C., Dunn L., Aroutiounova N. (2013). Adverse cardiac remodeling due to maternal low protein diet is associated with alterations in expression of genes regulating glucose metabolism. Nutr. Metab. Cardiovasc. Dis..

[6] Menendez-Castro C., Toka O., Fahlbusch F., Cordasic N., Wachtveitl R., Hilgers K.F., Rascher W., Hartner A. (2014). Impaired myocardial performance in a normotensive rat model of intrauterine growth restriction. Pediatr. Res..

[7] Hales C.N., Barker D.J. (2001). The thrifty phenotype hypothesis. Br. Med. Bull..

[8] Barker D.J., Osmond C., Golding J., Kuh D., Wadsworth M.E. (1989). Growth in utero, blood pressure in childhood and adult life, and mortality from cardiovascular disease. BMJ.

[9] Barker D.J., Bull A.R., Osmond C., Simmonds S.J. (1990). Fetal and placental size and risk of hypertension in adult life. BMJ.

[10] Barker D.J., Martyn C.N. (1997). The fetal origins of hypertension. Adv. Nephrol. Necker Hosp..

[11] Eriksson J.G., Forsen T.J., Kajantie E., Osmond C., Barker D.J. (2007). Childhood growth and hypertension in later life. Hypertension.

[12] Barker D.J. (1995). Fetal origins of coronary heart disease. BMJ.

[13] Fall C.H., Vijayakumar M., Barker D.J., Osmond C., Duggleby S. (1995). Weight in infancy and prevalence of coronary heart disease in adult life. BMJ.

[14] Leon D.A., Lithell H.O., Vagero D., Koupilova I., Mohsen R., Berglund L., Lithell U.B., McKeigue P.M. (1998). Reduced fetal growth rate and increased risk of death from ischaemic heart disease: Cohort study of 15,000 swedish men and women born 1915–29. BMJ.

[15] Eriksson J.G., Forsen T., Tuomilehto J., Winter P.D., Osmond C., Barker D.J. (1999). Catch-up growth in childhood and death from coronary heart disease: Longitudinal study. BMJ.

[16] Eriksson J.G., Forsen T., Tuomilehto J., Osmond C., Barker D.J. (2001). Early growth and coronary heart disease in later life: Longitudinal study. BMJ.

[17] Barker D.J., Osmond C., Forsen T.J., Kajantie E., Eriksson J.G. (2005). Trajectories of growth among children who have coronary events as adults. N. Engl. J. Med..

[18] Boney C.M., Verma A., Tucker R., Vohr B.R. (2005). Metabolic syndrome in childhood: Association with birth weight, maternal obesity, and gestational diabetes mellitus. Pediatrics.

[19] Harville E.W., Srinivasan S., Chen W., Berenson G.S. (2012). Is the metabolic syndrome a “small baby” syndrome?: The bogalusa heart study. Metab. Syndr. Relat. Disord..

[20] Goh K.L., Shore A.C., Quinn M., Tooke J.E. (2001). Impaired microvascular vasodilatory function in 3-month-old infants of low birth weight. Diabetes Care.

[21] Barker D.J. (2005). The developmental origins of insulin resistance. Horm. Res..

[22] Lopes A.A., Port F.K. (1995). The low birth weight hypothesis as a plausible explanation for the black/white differences in hypertension, non-insulin-dependent diabetes, and end-stage renal disease. Am. J. Kidney Dis..

[23] Vaag A.A., Poulsen P., Feinberg M.R. (1998). Is low birth weight a risk factor for development of non-insulin-dependent diabetes mellitus?. Ugeskr. Laeger.

[24] White S.L., Perkovic V., Cass A., Chang C.L., Poulter N., Spector T., Haysom L., Craig J.C., Salmi I.A., Chadban S.J. (2009). Is low birth weight an antecedent of ckd in later life? A systemic review of observational studies. Am. J. Kidney Dis..

[25] Mackenzie H.S., Lawler E.V., Brenner B.M. (1996). Congenital oligonephropathy: The fetal flaw in essential hypertension?. Kidney Int. Suppl..

[26] Keller G., Zimmer G., Mall G., Ritz E., Amann K. (2003). Nephron number in patients with primary hypertension. N. Engl. J. Med..

[27] Forsdahl A. (1977). Are poor living conditions in childhood and adolescence an important risk factor for arteriosclerotic heart disease?. Br. J. Prev. Soc. Med..

[28] Williams D.R., Roberts S.J., Davies T.W. (1979). Deaths from ischaemic heart disease and infant mortality in england and wales. J. Epidemiol. Community Health.

[29] Barker D.J., Winter P.D., Osmond C., Margetts B., Simmonds S.J. (1989). Weight in infancy and death from ischaemic heart disease. Lancet.

[30] Valdez R., Athens M.A., Thompson G.H., Bradshaw B.S., Stern M.P. (1994). Birthweight and adult health outcomes in a biethnic population in the USA. Diabetologia.

[31] Law C.M., Shiell A.W. (1996). Is blood pressure inversely related to birth weight? The strength of evidence from a systemic review of the literature. J. Hypertens..

[32] Stein C.E., Fall C.H., Kumaran K., Osmond C., Cox V., Barker D.J. (1996). Fetal growth and coronary heart disease in south india. Lancet.

[33] Jones S.E., Nyengaard J.R. (1998). Low birth weight and cardiovascular disease: Myth or reality?. Curr. Opin. Lipidol..

[34] Frontini M.G., Srinivasan S.R., Xu J., Berenson G.S. (2004). Low birth weight and longitudinal trends of cardiovascular risk factor variables from childhood to adolescence: The bogalusa heart study. BMC Pediatr..

[35] Banci M., Saccucci P., Dofcaci A., Sansoni I., Magrini A., Bottini E., Gloria-Bottini F. (2009). Birth weight and coronary artery disease. The effect of gender and diabetes. Int. J. Biol. Sci..

[36] Yajnik C. (2000). Interactions of perturbations in intrauterine growth and growth during childhood on the risk of adult-onset disease. Proc. Nutr. Soc..

[37] Hemachandra A.H., Howards P.P., Furth S.L., Klebanoff M.A. (2007). Birth weight, postnatal growth, and risk for high blood pressure at 7 years of age: Results from the collaborative perinatal project. Pediatrics.

[38] Huang R.C., Burke V., Newnham J.P., Stanley F.J., Kendall G.E., Landau L.I., Oddy W.H., Blake K.V., Palmer L.J., Beilin L.J. (2007). Perinatal and childhood origins of cardiovascular disease. Int. J. Obes. (Lond.).

[39] Huxley R.R., Shiell A.W., Law C.M. (2000). The role of size at birth and postnatal catch-up growth in determining systolic blood pressure: A systematic review of the literature. J. Hypertens..

[40] Barker D.J., Thornburg K.L. (2013). Placental programming of chronic diseases, cancer and lifespan: A review. Placenta.

[41] Taylor S.J., Whincup P.H., Cook D.G., Papacosta O., Walker M. (1997). Size at birth and blood pressure: Cross sectional study in 8–11 year old children. BMJ.

[42] Yiu V., Buka S., Zurakowski D., McCormick M., Brenner B., Jabs K. (1999). Relationship between birthweight and blood pressure in childhood. Am. J. Kidney Dis..

[43] Blake K.V., Gurrin L.C., Evans S.F., Beilin L.J., Landau L.I., Stanley F.J., Newnham J.P. (2000). Maternal cigarette smoking during pregnancy, low birth weight and subsequent blood pressure in early childhood. Early Hum. Dev..

[44] Adair L., Dahly D. (2005). Developmental determinants of blood pressure in adults. Annu. Rev. Nutr..

[45] Law C.M., de Swiet M., Osmond C., Fayers P.M., Barker D.J., Cruddas A.M., Fall C.H. (1993). Initiation of hypertension in utero and its amplification throughout life. BMJ.

[46] Leon D.A., Koupilova I., Lithell H.O., Berglund L., Mohsen R., Vagero D., Lithell U.B., McKeigue P.M. (1996). Failure to realise growth potential in utero and adult obesity in relation to blood pressure in 50 year old swedish men. BMJ.

[47] Koupilova I., Leon D.A., Lithell H.O., Berglund L. (1997). Size at birth and hypertension in longitudinally followed 50–70-year-old men. Blood Press..

[48] Curhan G.C., Willett W.C., Rimm E.B., Spiegelman D., Ascherio A.L., Stampfer M.J. (1996). Birth weight and adult hypertension, diabetes mellitus, and obesity in us men. Circulation.

[49] Uiterwaal C.S., Anthony S., Launer L.J., Witteman J.C., Trouwborst A.M., Hofman A., Grobbee D.E. (1997). Birth weight, growth, and blood pressure: An annual follow-up study of children aged 5 through 21 years. Hypertension.

[50] Leon D.A., Johansson M., Rasmussen F. (2000). Gestational age and growth rate of fetal mass are inversely associated with systolic blood pressure in young adults: An epidemiologic study of 165,136 swedish men aged 18 years. Am. J. Epidemiol..

[51] Hales C.N., Ozanne S.E. (2003). The dangerous road of catch-up growth. J. Physiol..

[52] Singhal A., Cole T.J., Fewtrell M., Deanfield J., Lucas A. (2004). Is slower early growth beneficial for long-term cardiovascular health?. Circulation.

[53] Lucas A. (1998). Programming by early nutrition: An experimental approach. J. Nutr..

[54] Singhal A., Lucas A. (2004). Early origins of cardiovascular disease: Is there a unifying hypothesis?. Lancet.

[55] Lucas A., Fewtrell M.S., Cole T.J. (1999). Fetal origins of adult disease-the hypothesis revisited. BMJ.

[56] Law C.M., Shiell A.W., Newsome C.A., Syddall H.E., Shinebourne E.A., Fayers P.M., Martyn C.N., de Swiet M. (2002). Fetal, infant, and childhood growth and adult blood pressure: A longitudinal study from birth to 22 years of age. Circulation.

[57] Jarvelin M.R., Sovio U., King V., Lauren L., Xu B., McCarthy M.I., Hartikainen A.L., Laitinen J., Zitting P., Rantakallio P. (2004). Early life factors and blood pressure at age 31 years in the 1966 northern finland birth cohort. Hypertension.

[58] Estourgie-van Burk G.F., Bartels M., Hoekstra R.A., Polderman T.J., Delemarre-van de Waal H.A., Boomsma D.I. (2009). A twin study of cognitive costs of low birth weight and catch-up growth. J. Pediatr..

[59] Crispi F., Hernandez-Andrade E., Pelsers M.M., Plasencia W., Benavides-Serralde J.A., Eixarch E., Le Noble F., Ahmed A., Glatz J.F., Nicolaides K.H. (2008). Cardiac dysfunction and cell damage across clinical stages of severity in growth-restricted fetuses. Am. J. Obstet. Gynecol..

[60] Kaijser M., Bonamy A.K., Akre O., Cnattingius S., Granath F., Norman M., Ekbom A. (2008). Perinatal risk factors for ischemic heart disease: Disentangling the roles of birth weight and preterm birth. Circulation.

[61] Crispi F., Bijnens B., Figueras F., Bartrons J., Eixarch E., Le Noble F., Ahmed A., Gratacos E. (2010). Fetal growth restriction results in remodeled and less efficient hearts in children. Circulation.

[62] Soto N., Bazaes R.A., Pena V., Salazar T., Avila A., Iniguez G., Ong K.K., Dunger D.B., Mericq M.V. (2003). Insulin sensitivity and secretion are related to catch-up growth in small-for-gestational-age infants at age 1 year: Results from a prospective cohort. J. Clin. Endocrinol. Metab..

[63] Forsen T., Eriksson J., Tuomilehto J., Reunanen A., Osmond C., Barker D. (2000). The fetal and childhood growth of persons who develop type 2 diabetes. Ann. Intern. Med..

[64] Ong K.K., Ahmed M.L., Emmett P.M., Preece M.A., Dunger D.B. (2000). Association between postnatal catch-up growth and obesity in childhood: Prospective cohort study. BMJ.

[65] Eriksson J., Forsen T., Tuomilehto J., Osmond C., Barker D. (2000). Fetal and childhood growth and hypertension in adult life. Hypertension.

[66] Fagerberg B., Bondjers L., Nilsson P. (2004). Low birth weight in combination with catch-up growth predicts the occurrence of the metabolic syndrome in men at late middle age: The atherosclerosis and insulin resistance study. J. Intern. Med..

[67] Valman H.B. (1979). Infants of low birth weight. Br. Med. J..

[68] Luke B., Petrie R.H. (1980). Intrauterine growth: Correlation of infant birth weight and maternal postpartum weight. Am. J. Clin. Nutr..

[69] Kiely J.L., Susser M. (1992). Preterm birth, intrauterine growth retardation, and perinatal mortality. Am. J. Public Health.

[70] Resnik R. (2002). Intrauterine growth restriction. Obstet. Gynecol..

[71] Wollmann H.A. (1998). Intrauterine growth restriction: Definition and etiology. Horm. Res..

[72] Lockwood C.J., Weiner S. (1986). Assessment of fetal growth. Clin. Perinatol..

[73] Malamitsi-Puchner A., Nikolaou K.E., Puchner K.P. (2006). Intrauterine growth restriction, brain-sparing effect, and neurotrophins. Ann. N. Y. Acad. Sci..

[74] Beeby A.R., Dunlop W., Hunter S. (1989). Evidence of redistribution of cardiac output in asymmetrical growth retardation. Br. J. Obstet. Gynaecol..

[75] Patterson R.M., Pouliot M.R. (1987). Neonatal morphometrics and perinatal outcome: Who is growth retarded?. Am. J. Obstet. Gynecol..

[76] Peleg D., Kennedy C.M., Hunter S.K. (1998). Intrauterine growth restriction: Identification and management. Am. Fam. Physician.

[77] Zadik Z. (2003). Maternal nutrition, fetal weight, body composition and disease in later life. J. Endocrinol. Investig..

[78] Widdowson E.M. (1974). Trace elements in foetal and early postnatal development. Proc. Nutr. Soc..

[79] Bergmann R.L., Bergmann K.E., Dudenhausen J.W. (2008). Undernutrition and growth restriction in pregnancy. Nestle Nutr. Workshop Ser. Pediatr. Program.

[80] Barker D.J. (1994). Outcome of low birthweight. Horm. Res..

[81] Barker D.J., Bergmann R.L., Ogra P.L. (2008). Concluding remarks. The window of opportunity: Pre-pregnancy to 24 months of age. Nestle Nutr. Workshop Ser. Pediatr. Program.

[82] Desai M., Crowther N.J., Lucas A., Hales C.N. (1996). Organ-selective growth in the offspring of protein-restricted mothers. Br. J. Nutr..

[83] Crews F., He J., Hodge C. (2007). Adolescent cortical development: A critical period of vulnerability for addiction. Pharmacol. Biochem. Behav..

[84] Gluckman P.D., Hanson M.A., Low F.M. (2011). The role of developmental plasticity and epigenetics in human health. Birth Defects Res. C Embryo Today.

[85] Godfrey K.M., Barker D.J. (2000). Fetal nutrition and adult disease. Am. J. Clin. Nutr..

[86] Symonds M.E., Sebert S.P., Hyatt M.A., Budge H. (2009). Nutritional programming of the metabolic syndrome. Nat. Rev. Endocrinol..

[87] Lucas A. (1991). Programming by early nutrition in man. Ciba Found. Symp..

[88] Godfrey K.M., Barker D.J. (1995). Maternal nutrition in relation to fetal and placental growth. Eur. J. Obstet. Gynecol. Reprod. Biol..

[89] Langley-Evans S.C. (2006). Developmental programming of health and disease. Proc. Nutr. Soc..

[90] Gluckman P.D., Cutfield W., Hofman P., Hanson M.A. (2005). The fetal, neonatal, and infant environments-the long-term consequences for disease risk. Early Hum. Dev..

[91] Edwards L.J., McFarlane J.R., Kauter K.G., McMillen I.C. (2005). Impact of periconceptional nutrition on maternal and fetal leptin and fetal adiposity in singleton and twin pregnancies. Am. J. Physiol. Regul. Integr. Comp. Physiol..

[92] Edwards L.J., McMillen I.C. (2002). Periconceptional nutrition programs development of the cardiovascular system in the fetal sheep. Am. J. Physiol. Regul. Integr. Comp. Physiol..

[93] Woods L.L., Weeks D.A., Rasch R. (2004). Programming of adult blood pressure by maternal protein restriction: Role of nephrogenesis. Kidney Int..

[94] Zimanyi M.A., Denton K.M., Forbes J.M., Thallas-Bonke V., Thomas M.C., Poon F., Black M.J. (2006). A developmental nephron deficit in rats is associated with increased susceptibility to a secondary renal injury due to advanced glycation end-products. Diabetologia.

[95] Hoppe C.C., Evans R.G., Bertram J.F., Moritz K.M. (2007). Effects of dietary protein restriction on nephron number in the mouse. Am. J. Physiol. Regul. Integr. Comp. Physiol..

[96] Briscoe T.A., Rehn A.E., Dieni S., Duncan J.R., Wlodek M.E., Owens J.A., Rees S.M. (2004). Cardiovascular and renal disease in the adolescent guinea pig after chronic placental insufficiency. Am. J. Obstet. Gynecol..

[97] Bassan H., Trejo L.L., Kariv N., Bassan M., Berger E., Fattal A., Gozes I., Harel S. (2000). Experimental intrauterine growth retardation alters renal development. Pediatr. Nephrol..

[98] Zohdi V., Moritz K.M., Bubb K.J., Cock M.L., Wreford N., Harding R., Black M.J. (2007). Nephrogenesis and the renal renin-angiotensin system in fetal sheep: Effects of intrauterine growth restriction during late gestation. Am. J. Physiol. Regul. Integr. Comp. Physiol..

[99] Corstius H.B., Zimanyi M.A., Maka N., Herath T., Thomas W., van der Laarse A., Wreford N.G., Black M.J. (2005). Effect of intrauterine growth restriction on the number of cardiomyocytes in rat hearts. Pediatr. Res..

[100] Stacy V., de Matteo R., Brew N., Sozo F., Probyn M.E., Harding R., Black M.J. (2009). The influence of naturally occurring differences in birthweight on ventricular cardiomyocyte number in sheep. Anat. Rec. (Hoboken).

[101] Bedi K.S., Birzgalis A.R., Mahon M., Smart J.L., Wareham A.C. (1982). Early life undernutrition in rats. 1. Quantitative histology of skeletal muscles from underfed young and refed adult animals. Br. J. Nutr..

[102] Tilley R.E., McNeil C.J., Ashworth C.J., Page K.R., McArdle H.J. (2007). Altered muscle development and expression of the insulin-like growth factor system in growth retarded fetal pigs. Domest. Anim. Endocrinol..

[103] Fahey A.J., Brameld J.M., Parr T., Buttery P.J. (2005). The effect of maternal undernutrition before muscle differentiation on the muscle fiber development of the newborn lamb. J. Anim. Sci..

[104] Snoeck A., Remacle C., Reusens B., Hoet J.J. (1990). Effect of a low protein diet during pregnancy on the fetal rat endocrine pancreas. Biol. Neonate.

[105] Vonnahme K.A., Zhu M.J., Borowicz P.P., Geary T.W., Hess B.W., Reynolds L.P., Caton J.S., Means W.J., Ford S.P. (2007). Effect of early gestational undernutrition on angiogenic factor expression and vascularity in the bovine placentome. J. Anim. Sci..

[106] Langley-Evans S.C., Gardner D.S., Jackson A.A. (1996). Maternal protein restriction influences the programming of the rat hypothalamic-pituitary-adrenal axis. J. Nutr..

[107] Leonhardt M., Lesage J., Dufourny L., Dickes-Coopman A., Montel V., Dupouy J.P. (2002). Perinatal maternal food restriction induces alterations in hypothalamo-pituitary-adrenal axis activity and in plasma corticosterone-binding globulin capacity of weaning rat pups. Neuroendocrinology.

[108] Brans Y.W., Kuehl T.J., Hayashi R.H., Andrew D.S., Reyes P. (1986). Amniotic fluid in baboon pregnancies with normal* versus* growth-retarded fetuses. Am. J. Obstet. Gynecol..

[109] Oyama K., Padbury J., Chappell B., Martinez A., Stein H., Humme J. (1992). Single umbilical artery ligation-induced fetal growth retardation: Effect on postnatal adaptation. Am. J. Physiol..

[110] Cock M.L., Harding R. (1997). Renal and amniotic fluid responses to umbilicoplacental embolization for 20 days in fetal sheep. Am. J. Physiol..

[111] Louey S., Cock M.L., Stevenson K.M., Harding R. (2000). Placental insufficiency and fetal growth restriction lead to postnatal hypotension and altered postnatal growth in sheep. Pediatr. Res..

[112] Cock M.L., Joyce B.J., Hooper S.B., Wallace M.J., Gagnon R., Brace R.A., Louey S., Harding R. (2004). Pulmonary elastin synthesis and deposition in developing and mature sheep: Effects of intrauterine growth restriction. Exp. Lung Res..

[113] Mitchell E.K., Louey S., Cock M.L., Harding R., Black M.J. (2004). Nephron endowment and filtration surface area in the kidney after growth restriction of fetal sheep. Pediatr. Res..

[114] Bubb K.J., Cock M.L., Black M.J., Dodic M., Boon W.M., Parkington H.C., Harding R., Tare M. (2007). Intrauterine growth restriction delays cardiomyocyte maturation and alters coronary artery function in the fetal sheep. J. Physiol..

[115] Merlet-Benichou C., Gilbert T., Muffat-Joly M., Lelievre-Pegorier M., Leroy B. (1994). Intrauterine growth retardation leads to a permanent nephron deficit in the rat. Pediatr. Nephrol..

[116] Langley-Evans S.C., Welham S.J., Jackson A.A. (1999). Fetal exposure to a maternal low protein diet impairs nephrogenesis and promotes hypertension in the rat. Life Sci..

[117] Zimanyi M.A., Bertram J.F., Black M.J. (2004). Does a nephron deficit in rats predispose to salt-sensitive hypertension?. Kidney Blood Press. Res..

[118] Cheema K.K., Dent M.R., Saini H.K., Aroutiounova N., Tappia P.S. (2005). Prenatal exposure to maternal undernutrition induces adult cardiac dysfunction. Br. J. Nutr..

[119] Langley-Evans S.C. (2014). Nutrition in early life and the programming of adult disease: A review. J. Hum. Nutr. Diet..

[120] Turner M.R. (1973). Perinatal mortality, growth and survival to weaning in offspring of rats reared on diets moderately deficient in protein. Br. J. Nutr..

[121] Ozanne S.E., Wang C.L., Coleman N., Smith G.D. (1996). Altered muscle insulin sensitivity in the male offspring of protein-malnourished rats. Am. J. Physiol..

[122] Woods L.L., Ingelfinger J.R., Nyengaard J.R., Rasch R. (2001). Maternal protein restriction suppresses the newborn renin-angiotensin system and programs adult hypertension in rats. Pediatr. Res..

[123] Bellinger L., Sculley D.V., Langley-Evans S.C. (2006). Exposure to undernutrition in fetal life determines fat distribution, locomotor activity and food intake in ageing rats. Int. J. Obes. (Lond.).

[124] Plank C., Ostreicher I., Hartner A., Marek I., Struwe F.G., Amann K., Hilgers K.F., Rascher W., Dotsch J. (2006). Intrauterine growth retardation aggravates the course of acute mesangioproliferative glomerulonephritis in the rat. Kidney Int..

[125] Alwasel S.H., Ashton N. (2009). Prenatal programming of renal sodium handling in the rat. Clin. Sci. (Lond.).

[126] Boubred F., Daniel L., Buffat C., Feuerstein J.M., Tsimaratos M., Oliver C., Dignat-George F., Lelievre-Pegorier M., Simeoni U. (2009). Early postnatal overfeeding induces early chronic renal dysfunction in adult male rats. Am. J. Physiol. Renal Physiol..

[127] Sherman R.C., Langley-Evans S.C. (2000). Antihypertensive treatment in early postnatal life modulates prenatal dietary influences upon blood pressure in the rat. Clin. Sci. (Lond.).

[128] Swali A., McMullen S., Langley-Evans S.C. (2010). Prenatal protein restriction leads to a disparity between aortic and peripheral blood pressure in wistar male offspring. J. Physiol..

[129] Kwong W.Y., Wild A.E., Roberts P., Willis A.C., Fleming T.P. (2000). Maternal undernutrition during the preimplantation period of rat development causes blastocyst abnormalities and programming of postnatal hypertension. Development.

[130] Manning J., Beutler K., Knepper M.A., Vehaskari V.M. (2002). Upregulation of renal bsc1 and tsc in prenatally programmed hypertension. Am. J. Physiol. Renal Physiol..

[131] Sathishkumar K., Elkins R., Yallampalli U., Yallampalli C. (2009). Protein restriction during pregnancy induces hypertension and impairs endothelium-dependent vascular function in adult female offspring. J. Vasc. Res..

[132] Dagan A., Habib S., Gattineni J., Dwarakanath V., Baum M. (2009). Prenatal programming of rat thick ascending limb chloride transport by low-protein diet and dexamethasone. Am. J. Physiol. Regul. Integr. Comp. Physiol..

[133] Habib S., Gattineni J., Twombley K., Baum M. (2011). Evidence that prenatal programming of hypertension by dietary protein deprivation is mediated by fetal glucocorticoid exposure. Am. J. Hypertens..

[134] Langley S.C., Jackson A.A. (1994). Increased systolic blood pressure in adult rats induced by fetal exposure to maternal low protein diets. Clin. Sci. (Lond.).

[135] Manning J., Vehaskari V.M. (2001). Low birth weight-associated adult hypertension in the rat. Pediatr. Nephrol..

[136] Tonkiss J., Trzcinska M., Galler J.R., Ruiz-Opazo N., Herrera V.L. (1998). Prenatal malnutrition-induced changes in blood pressure: Dissociation of stress and nonstress responses using radiotelemetry. Hypertension.

[137] Vehaskari V.M., Aviles D.H., Manning J. (2001). Prenatal programming of adult hypertension in the rat. Kidney Int..

[138] Coupe B., Grit I., Darmaun D., Parnet P. (2009). The timing of “catch-up growth” affects metabolism and appetite regulation in male rats born with intrauterine growth restriction. Am. J. Physiol. Regul. Integr. Comp. Physiol..

[139] Plank C., Grillhosl C., Ostreicher I., Meissner U., Struwe F.G., Rauh M., Hartner A., Rascher W., Dotsch J. (2008). Transient growth hormone therapy to rats with low protein-inflicted intrauterine growth restriction does not prevent elevated blood pressure in later life. Growth Factors.

[140] Zeng Y., Gu P., Liu K., Huang P. (2013). Maternal protein restriction in rats leads to reduced pgc-1alpha expression via altered DNA methylation in skeletal muscle. Mol. Med. Rep..

[141] Menendez-Castro C., Fahlbusch F., Cordasic N., Amann K., Munzel K., Plank C., Wachtveitl R., Rascher W., Hilgers K.F., Hartner A. (2011). Early and late postnatal myocardial and vascular changes in a protein restriction rat model of intrauterine growth restriction. PLoS One.

[142] Woods L.L., Ingelfinger J.R., Rasch R. (2005). Modest maternal protein restriction fails to program adult hypertension in female rats. Am. J. Physiol. Regul. Integr. Comp. Physiol..

[143] Lim K., Zimanyi M.A., Black M.J. (2006). Effect of maternal protein restriction in rats on cardiac fibrosis and capillarization in adulthood. Pediatr. Res..

[144] Lim K., Zimanyi M.A., Black M.J. (2010). Effect of maternal protein restriction during pregnancy and lactation on the number of cardiomyocytes in the postproliferative weanling rat heart. Anat. Rec. (Hoboken).

[145] Lim K., Armitage J.A., Stefanidis A., Oldfield B.J., Black M.J. (2011). IUGR in the absence of postnatal “catch-up” growth leads to improved whole body insulin sensitivity in rat offspring. Pediatr. Res..

[146] Lim K., Lombardo P., Schneider-Kolsky M., Hilliard L., Denton K.M., Black M.J. (2011). Induction of hyperglycemia in adult intrauterine growth-restricted rats: Effects on renal function. Am. J. Physiol. Renal. Physiol..

[147] Lim K., Lombardo P., Schneider-Kolsky M., Black M.J. (2012). Intrauterine growth restriction coupled with hyperglycemia: Effects on cardiac structure in adult rats. Pediatr. Res..

[148] Zohdi V., Jane Black M., Pearson J.T. (2011). Elevated vascular resistance and afterload reduce the cardiac output response to dobutamine in early growth-restricted rats in adulthood. Br. J. Nutr..

[149] Zohdi V., Wood B.R., Pearson J.T., Bambery K.R., Black M.J. (2013). Evidence of altered biochemical composition in the hearts of adult intrauterine growth-restricted rats. Eur. J. Nutr..

[150] Zohdi V., Pearson J.T., Kett M.M., Lombardo P., Schneider M., Black M.J. (2014). When early life growth restriction in rats is followed by attenuated postnatal growth: Effects on cardiac function in adulthood. Eur. J. Nutr..

[151] Brawley L., Itoh S., Torrens C., Barker A., Bertram C., Poston L., Hanson M. (2003). Dietary protein restriction in pregnancy induces hypertension and vascular defects in rat male offspring. Pediatr. Res..

[152] Elmes M.J., Gardner D.S., Langley-Evans S.C. (2007). Fetal exposure to a maternal low-protein diet is associated with altered left ventricular pressure response to ischaemia-reperfusion injury. Br. J. Nutr..

[153] Gardner D.S., Jackson A.A., Langley-Evans S.C. (1997). Maintenance of maternal diet-induced hypertension in the rat is dependent on glucocorticoids. Hypertension.

[154] Harrison M., Langley-Evans S.C. (2009). Intergenerational programming of impaired nephrogenesis and hypertension in rats following maternal protein restriction during pregnancy. Br. J. Nutr..

[155] Langley-Evans S.C., Phillips G.J., Jackson A.A. (1994). In utero exposure to maternal low protein diets induces hypertension in weanling rats, independently of maternal blood pressure changes. Clin. Nutr..

[156] Langley-Evans S.C., Phillips G.J., Benediktsson R., Gardner D.S., Edwards C.R., Jackson A.A., Seckl J.R. (1996). Protein intake in pregnancy, placental glucocorticoid metabolism and the programming of hypertension in the rat. Placenta.

[157] McMullen S., Langley-Evans S.C. (2005). Maternal low-protein diet in rat pregnancy programs blood pressure through sex-specific mechanisms. Am. J. Physiol. Regul. Integr. Comp. Physiol..

[158] McMullen S., Gardner D.S., Langley-Evans S.C. (2004). Prenatal programming of angiotensin ii type 2 receptor expression in the rat. Br. J. Nutr..

[159] Mehta G., Roach H.I., Langley-Evans S., Taylor P., Reading I., Oreffo R.O., Aihie-Sayer A., Clarke N.M., Cooper C. (2002). Intrauterine exposure to a maternal low protein diet reduces adult bone mass and alters growth plate morphology in rats. Calcif. Tissue Int..

[160] Nwagwu M.O., Cook A., Langley-Evans S.C. (2000). Evidence of progressive deterioration of renal function in rats exposed to a maternal low-protein diet in utero. Br. J. Nutr..

[161] Pladys P., Sennlaub F., Brault S., Checchin D., Lahaie I., Le N.L., Bibeau K., Cambonie G., Abran D., Brochu M. (2005). Microvascular rarefaction and decreased angiogenesis in rats with fetal programming of hypertension associated with exposure to a low-protein diet in utero. Am. J. Physiol. Regul. Integr. Comp. Physiol..

[162] Tappia P.S., Nijjar M.S., Mahay A., Aroutiounova N., Dhalla N.S. (2005). Phospholipid profile of developing heart of rats exposed to low-protein diet in pregnancy. Am. J. Physiol. Regul. Integr. Comp. Physiol..

[163] Tappia P.S., Thliveris J., Xu Y.J., Aroutiounova N., Dhalla N.S. (2011). Effects of amino acid supplementation on myocardial cell damage and cardiac function in diabetes. Exp. Clin. Cardiol..

[164] Torrens C., Brawley L., Anthony F.W., Dance C.S., Dunn R., Jackson A.A., Poston L., Hanson M.A. (2006). Folate supplementation during pregnancy improves offspring cardiovascular dysfunction induced by protein restriction. Hypertension.

[165] Ohishi T., Wang L., Akane H., Shiraki A., Sato A., Uematsu M., Suzuki K., Mitsumori K., Shibutani M. (2012). Adolescent hyperactivity of offspring after maternal protein restriction during the second half of gestation and lactation periods in rats. J. Toxicol. Sci..

[166] Reyes-Castro L.A., Rodriguez J.S., Rodriguez-Gonzalez G.L., Wimmer R.D., McDonald T.J., Larrea F., Nathanielsz P.W., Zambrano E. (2011). Pre- and/or postnatal protein restriction in rats impairs learning and motivation in male offspring. Int. J. Dev. Neurosci..

[167] Zambrano E., Bautista C.J., Deas M., Martinez-Samayoa P.M., Gonzalez-Zamorano M., Ledesma H., Morales J., Larrea F., Nathanielsz P.W. (2006). A low maternal protein diet during pregnancy and lactation has sex- and window of exposure-specific effects on offspring growth and food intake, glucose metabolism and serum leptin in the rat. J. Physiol..

[168] Li F., Wang X., Capasso J.M., Gerdes A.M. (1996). Rapid transition of cardiac myocytes from hyperplasia to hypertrophy during postnatal development. J. Mol. Cell. Cardiol..

[169] Oliver M.H., Hawkins P., Harding J.E. (2005). Periconceptional undernutrition alters growth trajectory and metabolic and endocrine responses to fasting in late-gestation fetal sheep. Pediatr. Res..

[170] Oliver M.H., Jaquiery A.L., Bloomfield F.H., Harding J.E. (2007). The effects of maternal nutrition around the time of conception on the health of the offspring. Soc. Reprod. Fertil. Suppl..

[171] Chavatte-Palmer P., Al Gubory K., Picone O., Heyman Y. (2008). Maternal nutrition: Effects on offspring fertility and importance of the periconceptional period on long-term development. Gynecol. Obstet. Fertil..

[172] Zhang S., Rattanatray L., McMillen I.C., Suter C.M., Morrison J.L. (2011). Periconceptional nutrition and the early programming of a life of obesity or adversity. Prog. Biophys. Mol. Biol..

[173] McMillen I.C., MacLaughlin S.M., Muhlhausler B.S., Gentili S., Duffield J.L., Morrison J.L. (2008). Developmental origins of adult health and disease: The role of periconceptional and foetal nutrition. Basic Clin. Pharmacol. Toxicol..

[174] Rumball C.W., Bloomfield F.H., Oliver M.H., Harding J.E. (2009). Different periods of periconceptional undernutrition have different effects on growth, metabolic and endocrine status in fetal sheep. Pediatr. Res..

[175] Gamborg M., Byberg L., Rasmussen F., Andersen P.K., Baker J.L., Bengtsson C., Canoy D., Droyvold W., Eriksson J.G., Forsen T. (2007). Birth weight and systolic blood pressure in adolescence and adulthood: Meta-regression analysis of sex- and age-specific results from 20 nordic studies. Am. J. Epidemiol..

[176] Chen W., Srinivasan S.R., Berenson G.S. (2010). Amplification of the association between birthweight and blood pressure with age: The bogalusa heart study. J. Hypertens..

[177] Chen W., Srinivasan S.R., Ruan L., Mei H., Berenson G.S. (2011). Adult hypertension is associated with blood pressure variability in childhood in blacks and whites: The bogalusa heart study. Am. J. Hypertens..

[178] O’Regan D., Kenyon C.J., Seckl J.R., Holmes M.C. (2008). Prenatal dexamethasone “programmes” hypotension, but stress-induced hypertension in adult offspring. J. Endocrinol..

[179] Gluckman P.D., Hanson M.A. (2004). The developmental origins of the metabolic syndrome. Trends Endocrinol. Metab..

[180] Armitage J.A., Khan I.Y., Taylor P.D., Nathanielsz P.W., Poston L. (2004). Developmental programming of the metabolic syndrome by maternal nutritional imbalance: How strong is the evidence from experimental models in mammals?. J. Physiol..

[181] McMillen I.C., Robinson J.S. (2005). Developmental origins of the metabolic syndrome: Prediction, plasticity, and programming. Physiol. Rev..

[182] Stocker C.J., Arch J.R., Cawthorne M.A. (2005). Fetal origins of insulin resistance and obesity. Proc. Nutr. Soc..

[183] Gluckman P.D., Hanson M.A., Cooper C., Thornburg K.L. (2008). Effect of in utero and early-life conditions on adult health and disease. N. Engl. J. Med..

[184] Augustyniak R.A., Singh K., Zeldes D., Singh M., Rossi N.F. (2010). Maternal protein restriction leads to hyperresponsiveness to stress and salt-sensitive hypertension in male offspring. Am. J. Physiol. Regul. Integr. Comp. Physiol..

[185] Van Abeelen A.F., Veenendaal M.V., Painter R.C., de Rooij S.R., Thangaratinam S., van Der Post J.A., Bossuyt P.M., Elias S.G., Uiterwaal C.S., Grobbee D.E. (2012). The fetal origins of hypertension: A systematic review and meta-analysis of the evidence from animal experiments of maternal undernutrition. J. Hypertens..

[186] Grassi G., Facchetti R., Seravalle G., Cuspidi C., Mancia G. (2013). Home and ambulatory blood pressure in resistant hypertension. EuroIntervention.

[187] Mancia G., Bombelli M., Brambilla G., Facchetti R., Sega R., Toso E., Grassi G. (2013). Long-term prognostic value of white coat hypertension: An insight from diagnostic use of both ambulatory and home blood pressure measurements. Hypertension.

[188] Martin U., Holder R., Hodgkinson J., McManus R. (2013). Inter-arm blood pressure differences compared with ambulatory monitoring: A manifestation of the “white-coat” effect?. Br. J. Gen. Pract..

[189] R Rios M.T., Dominguez-Sardina M., Ayala D.E., Gomara S., Sineiro E., Pousa L., Callejas P.A., Fontao M.J., Fernandez J.R., Hermida R.C. (2013). Prevalence and clinical characteristics of isolated-office and true resistant hypertension determined by ambulatory blood pressure monitoring. Chronobiol. Int..

[190] Sung S.H., Cheng H.M., Wang K.L., Yu W.C., Chuang S.Y., Ting C.T., Lakatta E.G., Yin F.C., Chou P., Chen C.H. (2013). White coat hypertension is more risky than prehypertension: Important role of arterial wave reflections. Hypertension.

[191] Wight T.N. (1989). Cell biology of arterial proteoglycans. Arteriosclerosis.

[192] Halper J. (2014). Proteoglycans and diseases of soft tissues. Adv. Exp. Med. Biol..

[193] Ozanne S.E., Hales C.N. (2002). Early programming of glucose-insulin metabolism. Trends Endocrinol. Metab..

[194] Fagundes A.T., Moura E.G., Passos M.C., Santos-Silva A.P., de Oliveira E., Trevenzoli I.H., Casimiro-Lopes G., Nogueira-Neto J.F., Lisboa P.C. (2009). Temporal evaluation of body composition, glucose homeostasis and lipid profile of male rats programmed by maternal protein restriction during lactation. Horm. Metab. Res..

[195] Devereux R.B., Roman M.J. (1999). Left ventricular hypertrophy in hypertension: Stimuli, patterns, and consequences. Hypertens. Res..

[196] Palmieri V., Bella J.N., DeQuattro V., Roman M.J., Hahn R.T., Dahlof B., Sharpe N., Lau C.P., Chen W.C., Paran E. (1999). Relations of diastolic left ventricular filling to systolic chamber and myocardial contractility in hypertensive patients with left ventricular hypertrophy (the preserve study). Am. J. Cardiol..

[197] Elnakish M.T., Hassanain H.H., Janssen P.M. (2012). Vascular remodeling-associated hypertension leads to left ventricular hypertrophy and contractile dysfunction in profilin-1 transgenic mice. J. Cardiovasc. Pharmacol..

[198] Sanchez-Soria P., Broka D., Monks S.L., Camenisch T.D. (2012). Chronic low-level arsenite exposure through drinking water increases blood pressure and promotes concentric left ventricular hypertrophy in female mice. Toxicol. Pathol..

[199] Nadruz W. (2014). Myocardial remodeling in hypertension. J. Hum. Hypertens..

[200] Sampson A.K., Moritz K.M., Jones E.S., Flower R.L., Widdop R.E., Denton K.M. (2008). Enhanced angiotensin ii type 2 receptor mechanisms mediate decreases in arterial pressure attributable to chronic low-dose angiotensin ii in female rats. Hypertension.

[201] Payne J.A., Alexander B.T., Khalil R.A. (2004). Decreased endothelium-dependent no-cgmp vascular relaxation and hypertension in growth-restricted rats on a high-salt diet. Hypertension.

[202] Sanders M.W., Fazzi G.E., Janssen G.M., Blanco C.E., de Mey J.G. (2005). High sodium intake increases blood pressure and alters renal function in intrauterine growth-retarded rats. Hypertension.

[203] Myrie S.B., McKnight L.L., King J.C., McGuire J.J., van Vliet B.N., Bertolo R.F. (2012). Effects of a diet high in salt, fat, and sugar on telemetric blood pressure measurements in conscious, unrestrained adult yucatan miniature swine (sus scrofa). Comp. Med..

[204] Langley-Evans S.C., Jackson A.A. (1996). Rats with hypertension induced by in utero exposure to maternal low-protein diets fail to increase blood pressure in response to a high salt intake. Ann. Nutr. Metab..

[205] Yu H.C., Burrell L.M., Black M.J., Wu L.L., Dilley R.J., Cooper M.E., Johnston C.I. (1998). Salt induces myocardial and renal fibrosis in normotensive and hypertensive rats. Circulation.

[206] Whaley-Connell A.T., Habibi J., Aroor A., Ma L., Hayden M.R., Ferrario C.M., Demarco V.G., Sowers J.R. (2013). Salt loading exacerbates diastolic dysfunction and cardiac remodeling in young female ren2 rats. Metabolism.

